# Peritumoral edema status of glioblastoma identifies patients reaching long-term disease control with specific progression patterns after tumor resection and high-dose proton boost

**DOI:** 10.1007/s00432-021-03765-6

**Published:** 2021-08-30

**Authors:** Hsiang-Kuang Tony Liang, Masashi Mizumoto, Eiichi Ishikawa, Masahide Matsuda, Keiichi Tanaka, Hidehiro Kohzuki, Haruko Numajiri, Yoshiko Oshiro, Toshiyuki Okumura, Akira Matsumura, Hideyuki Sakurai

**Affiliations:** 1 Department of Biomedical Engineering, National Taiwan University, Taipei, Taiwan; 2 Department of Radiation Oncology, Proton Medical Research Center, University of Tsukuba, Tsukuba, Ibaraki Japan; 3 Department of Neurosurgery, Faculty of Medicine, University of Tsukuba, Tsukuba, Ibaraki Japan; 4 Department of Radiation Oncology, National Taiwan University Cancer Center, National Taiwan University Hospital, Taipei, Taiwan; 5 Division of Radiation Oncology, National Taiwan University Hospital, Taipei, Taiwan; 6 Department of Radiation Oncology, Tsukuba Medical Center Hospital, Tsukuba, Ibaraki Japan

**Keywords:** Glioblastoma, Peritumoral edema, Imaging biomarker, Dose-escalated radiotherapy, Proton beam therapy, Personalized treatment

## Abstract

**Background:**

Glioblastoma peritumoral edema (PE) extent is associated with survival and progression pattern after tumor resection and radiotherapy (RT). To increase tumor control, proton beam was adopted to give high-dose boost (> 90 Gy). However, the correlation between PE extent and prognosis of glioblastoma after postoperative high-dose proton boost (HDPB) therapy stays unknown. We intend to utilize the PE status to classify the survival and progression patterns.

**Methods:**

Patients receiving HDPB (96.6 GyE) were retrospectively evaluated. Limited peritumoral edema (LPE) was defined as PE extent < 3 cm with a ratio of PE extent to tumor maximum diameter of < 0.75. Extended progressive disease (EPD) was defined as progression of tumors extending > 1 cm from the tumor bed edge.

**Results:**

After long-term follow-up (median 88.7, range 63.6–113.8 months) for surviving patients with (n = 13) and without (n = 32) LPE, the median overall survival (OS) and progression-free survival (PFS) were 77.2 vs. 16.7 months (*p* = 0.004) and 13.6 vs. 8.6 months (*p* = 0.02), respectively. In multivariate analyses combined with factors of performance, age, tumor maximum diameter, and tumor resection extent, LPE remained a significant factor for favorable OS and PFS. The rates of 5-year complete response, EPD, and distant metastasis with and without LPE were 38.5% vs. 3.2% (*p* = 0.005), 7.7% vs. 40.6% (*p* = 0.04), and 0% vs. 34.4% (*p* = 0.02), respectively.

**Conclusions:**

The LPE status effectively identified patients with relative long-term control and specific progression patterns after postoperative HDPB for glioblastoma.

**Supplementary Information:**

The online version contains supplementary material available at 10.1007/s00432-021-03765-6.

## Introduction

Glioblastoma is the most common primary brain tumor in adults, and has median overall survival (OS) of 7–36 months, depending on the extent of peritumoral edema (PE), molecular biomarkers, age, and performance status (Stupp et al. 2009; Liang et al. 2017; Iuchi et al. 2014; Molenaar et al. 2014; Li et al. 2011; Mirimanoff et al. 2006). The extent of tumor resection is highly correlated with glioblastoma prognosis (Li et al. 2011; Mirimanoff et al. 2006). Perilesional resection (Al-Holou et al. 2019) or maximum safe resection of T1 contrast-enhancing tumors with or without surrounding abnormality in fluid-attenuated inversion recovery (FLAIR) images (Li et al. 2016) may also prolong survival. Radiotherapy (RT) is the standard of care to increase local control of glioblastoma after tumor resection (Kristiansen et al. 1981; Walker et al. 1978). However, with postoperative RT at a conventional dose (commonly 60 Gy), recurrence near the original tumor bed or within the irradiation field is the most frequent progression pattern (72–96.8%) (Brandes et al. 2009; Gebhardt et al. 2014; McDonald et al. 2011).

To increase disease control after tumor resection, RT with dose escalation or various fractionations has been investigated (Iuchi et al. 2014; Al-Holou et al. 2019; Li et al. 2016; Mizumoto et al. 2010; Navarria et al. 2017; Shenouda et al. 2017; Tanaka et al. 2005; Shrieve et al. 1999). In general, the 5-year complete response (CR) rates for glioblastoma after surgery followed by such RT is 0–1.6% (Stupp et al. 2009; Navarria et al. 2017; Shenouda et al. 2017; Tanaka et al. 2005). However, RT using dose escalation (> 60 Gy) is not commonly adopted for glioblastoma in clinical practice; thus, the population receiving RT at > 66 Gy is limited (about 4% in the US) and has diverse features (Wegner et al. 2019). Compared with photon irradiation, proton beams give a relatively conformal dose distribution to tumors and reduces the dose to normal tissues because of its Bragg peak effect (Adeberg et al. 2016). This facilitates a high-dose radiation boost to the glioblastoma tumor bed. Even though the use of proton beam therapy (PBT) is increasing worldwide, RT with high-dose proton boost (HDPB, > 90 Gy) to glioblastoma requires cautious investigation in phase I/II clinical trials (Mizumoto et al. 2010). Therefore, the patient population receiving tumor resection followed by RT of dose > 90 Gy is small.

The PE extent of glioblastoma is a well-accepted prognostic factor for survival after RT at a conventional dose (Liang et al. 2017; Schoenegger et al. 2009; Wu et al. 2015a). However, use of the PE status to classify prognosis after tumor resection followed by HDPB for glioblastoma has not been investigated. In particular, whether the PE status is able to classify survival and progression patterns of glioblastoma after HDPB remains unknown. From a pathophysiological perspective, PE of glioblastoma is correlated with effusion caused by blood–brain barrier damage (Wolburg et al. 2012) and cancer cell infiltration (Yamahara et al. 2010). From a radiological perspective, the extent and distribution of PE reveal the migratory ability of the tumor (Liang et al. 2017; Wu et al. 2015b). Glioblastomas with limited PE (LPE) are associated with more favorable survival and less tumor spreading than those with extensive PE for patients receiving RT of conventional dose (Liang et al. 2017; Wu et al. 2015a). Therefore, LPE cases should have longer disease control and less tumor spreading after postoperative HDPB, compared with those without LPE. Based on these pathophysiological and radiological perspectives, we integrated the PE extent into analysis of prognosis for glioblastoma after HDPB.

Utilization of the PE status to identify glioblastoma with probable long-term disease control after HDPB is crucial for future decision-making on use of PBT. Herein, we propose a novel hypothesis that the PE status of glioblastoma is an important prognostic factor for glioblastoma after tumor resection followed by HDPB. To verify this hypothesis, we analyzed clinical data for patients with glioblastoma who received HDPB (96.6 GyE) to determine the optimal cutoff value of LPE and then evaluated the association between LPE status and treatment outcomes, including survival and progression patterns. The overall goal of this study is to verify whether preoperative LPE status is effective to classify survival and progression patterns after HDPB, which should help personalize decision-making on strategies for glioblastoma treatment.

## Methods

### Study design

The retrospective study design adhered to the Strengthening the Reporting of Observational Studies in Epidemiology research reporting guidelines (Vandenbroucke et al. 2014). The treatment protocol of HDPB for glioblastoma was based on a previous phase I/II clinical trial (Mizumoto et al. 2010, 2015, 2016) approved by the Institutional Ethical Committee and Steering Committee. To identify patients with long-term disease-free survival after surgery and HDBP, the relationships of PE status and common prognostic factors with survival and progression patterns were analyzed. Statistical analysis was designed and performed through consultation with statisticians.

### Patient eligibility

Patients with pathologically diagnosed glioblastoma were eligible for HDPB after radiation oncologists and neurosurgeons confirmed that the predicted radiation necrosis was unlikely to be fatal (e.g., multifocal tumors or invasion to brainstem). Patients with tumors close to the optic chiasm, Karnofsky performance status (KPS) < 60 or age > 80 were also excluded. In total, 45 patients underwent surgery followed by HDPB from November 2001 to November 2012. Twenty patients were participants in a phase I/II clinical trial and additional 25 patients were recruited after confirming the safety and feasibility of the HDPB protocol. Written informed consent was obtained from all patients.

### Treatment methods

Maximum safe resection of gadolinium-enhanced lesions on T1-weighted images was performed for most patients and biopsy was used for those with unresectable lesions, after which all patients received concurrent chemoradiotherapy with HDPB. The tumor resection extent was classified as gross total resection (GTR), partial resection, and biopsy based on records of neurosurgeons and magnetic resonance imaging (MRI) scans after surgery. Nimustine was intravenously administered at 80 mg/m^2^ for 1 day in RT weeks one and four for patients treated before temozolomide became available. For those treated after use of temozolomide became possible, the chemotherapy regimen was uniformly shifted to temozolomide with daily oral administration at 75 mg/m^2^ (Stupp et al. 2002). After a 4-week break, patients received up to six cycles of adjuvant temozolomide for 5 days over a 28-day period and the dose was administered according to the treatment protocol proposed by Stupp et al. (2002). A MRI scan was performed at diagnosis and mainly within 72 h after surgery.

**Fig. 1:**
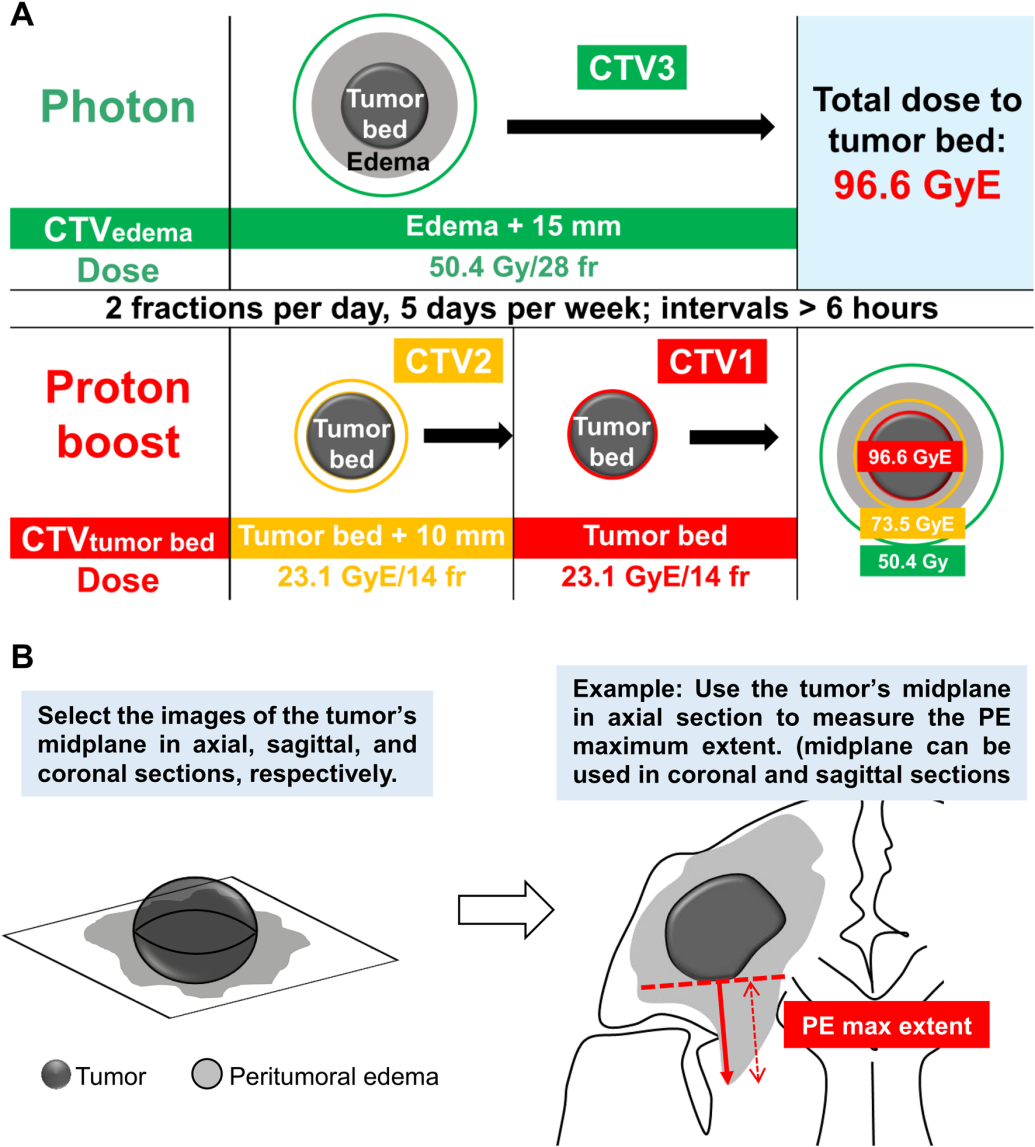
**A** Radiotherapy protocol in the current study. The radiotherapy courses, dose prescription and target definitions. **B** Method of measuring peritumoral edema extent in our study. First, we selected the images that presented the tumor’s midplane among axial, sagittal, and coronal sections, respectively. Then, we created tangential lines (red dash line) to the tumor edge and then measured the PE maximum extent from the tumor edge to the PE area edge along their normal lines (red line with arrowhead). *CTV* clinical target volume, *fr* fractions, *max* maximum, *PE* peritumoral edema

The tumor bed area comprised the surgical cavity and gadolinium-enhanced lesions on T1-weighted images. Clinical target volume (CTV)1 was defined as the tumor bed area, CTV2 as the tumor bed area plus a 10-mm margin, and CTV3 as hyperintense lesions on T2-weighted or FLAIR images plus a 15-mm margin. Figure 1A illustrates the RT courses and dose prescription used in the study. Large-field RT of 50.4 Gy was delivered in 28 fractions, primarily by photon, to CTV3 once per day in the morning for 5 days per week. When CTV3 of a tumor was not able to be irradiated by proton beam machine due to field size restriction, photon beam was adopted for CTV3 irradiation.

HDPB used hyperfractionated concomitant boost to the tumor bed by double scattering proton beams and was delivered on the same day with an interval of > 6 h after large-field RT. The relative biological effectiveness of the proton beam was taken to be 1.1 (Mizumoto et al. 2010). The first 23.1 GyE in 14 fractions was delivered to CTV2 and the other 23.1 GyE in 14 fractions was delivered to CTV1. The total radiation doses to CTV1, CTV2 and CTV3 were 96.6, 73.5, and 50.4 Gy, respectively. The planning target volume was defined as the CTV plus 5 mm for setup error. The estimated biological effective doses to targets (with associated equivalent doses in 2-Gy fractions, α/β = 10) for CTV1, CTV2, and CTV3 were 59 (50) Gy, 86 (72) Gy, and 113 (94) Gy, respectively (Machtay et al. 2012).

### Neuroimaging variables and definition of LPE

Neuroimaging features before surgery and RT were evaluated, including PE extent and tumor size. All MRI scans were reviewed by two radiation oncologists. PE extent and tumor size were assessed through integrating preoperative MRI sequences, including T2-weighted, FLAIR, and T1-weighted gadolinium-enhanced imaging. The preoperative T1-weighted gadolinium-enhanced lesion in MRI was defined as the tumor area. The hyperintense area in preoperative T2-weighted or FLAIR MRI outside the tumor area was defined as the PE after comparison with the T1-weighted gadolinium-enhanced lesion. We selected the images that presented the tumor’s midplane among axial, sagittal, and coronal sections to measure tumor maximum diameter and PE maximum extent, respectively. Tumor maximum diameter was determined by measuring the maximum length of tumor area among three sections (Chaichana et al. 2013; Raysi Dehcordi et al. 2012). PE maximum extent was determined by measuring the maximum PE length among three sections, which is illustrated in Fig. 1B (Liang et al. 2017). After determining these parameters, the edema-to-tumor ratio (ETR) was calculated by dividing the PE maximum extent by the tumor maximum diameter (Wangaryattawanich et al. 2015).

### Follow-up of tumor progression patterns after HDPB

The protocol for post-RT neuroimaging follow-up primarily consisted of MRI interpreted by neuroradiologists at intervals of about 3–4 months, adjusted to individual patient conditions. During follow-up, progressive disease (PD) was defined according to Response Assessment in Neuro-Oncology (RANO) criteria as (1) development of a new T1-weighted gadolinium-enhanced lesion, or (2) ≥ 25% enlargement of a T1-weighted gadolinium-enhanced lesion compared with the smallest tumor measured at best treatment response (Linhares et al. 2013; Wen et al. 2010). When suspected PD developed within 12 weeks after completion of RT, presentation with new enhancements beyond the radiation field helped differentiate PD from pseudoprogression (Linhares et al. 2013; Wen et al. 2010). Otherwise, serial imaging follow-up was performed until PD status in imaging was confirmed. The need for reoperation and pathological confirmation was determined by the neurosurgeon to differentiate radiation necrosis and pseudoprogression from tumor progression (Linhares et al. 2013; Wen et al. 2010). Salvage surgery with tumor resection was used for tumor progression based on patient performance status and tumor location. Limited PD and extended progressive disease (EPD) were defined as recurrent tumors continuously extending ≤ 1 and > 1 cm from the original tumor bed edge, respectively, compared with MRI immediately after surgery. PD patterns were classified as regional (involving the original peritumoral edematous areas, but not connecting with the original tumor area) and distant (not connecting to the original tumor or peritumoral edematous area) (Liang et al. 2016).

### Variables and statistical methods

Sensitivity analysis using an operating characteristic (ROC) curve was utilized to measure the ability of PE extent, tumor maximum diameter, ETR, age, and Karnofsky performance status (KPS) to discriminate between patients with and without CR for 5 years. In addition, different cutoff values of PE extent, tumor maximum diameters, and ETR according to ROC curve analysis results were used to classify survival. LPE was determined by integrating the PE extent and ETR variables based on the results of univariate survival and sensitivity analyses. To clarify potential bias or confounders in survival analysis for LPE, other common prognostic factors, including KPS, age, tumor maximum diameter, tumor resection extent, and chemotherapy regimen, were included in univariate and Cox regression survival analyses (Li et al. 2011; Mirimanoff et al. 2006; Chaichana et al. 2013; Raysi Dehcordi et al. 2012). The rates of common prognostic factors for patients according to LPE group and the significance of the association of LPE status with tumor progression patterns were examined by Fisher exact test.

OS was calculated from the date of first surgery to death. Progression-free survival (PFS) was calculated from the date of first surgery to that of disease progression, including death or PD confirmed on imaging. Patients who survived and were disease-free for 5 years from the date of first surgery were defined as cases with 5-year CR. Patients who were alive or lost to follow-up, but without tumor progression at the time of analysis, were censored cases. The Kaplan–Meier method was used for calculating survival, with between-group differences in survival compared by log-rank test. Survival data were based on HRs and 95% CIs. All tests were two-sided and the results were considered significant at *p* < 0.05. All calculations were performed using SPSS v.19 (SPSS, Chicago, IL, USA).

## Results

The subjects were 45 patients with glioblastoma treated with HDPB after surgery (30 GTR, 14 partial resection, and one biopsy). By April 2018, at a median follow-up period for surviving patients of 88.7 (range 63.6–113.8) months, 41 (91.1%) of the patients had disease progression, including 38 deaths (84.4%) and 34 cases with tumor progression (75.6%) based on radiological interpretation using RANO criteria (Linhares et al. 2013; Wen et al. 2010) or by pathological confirmation through reoperation. Among 17 patients receiving reoperation for pathology evaluation, 11 had tumor recurrence and six had radiation necrosis only.

### Survival after HDPB based on PE status

The results of a sensitivity analysis of 5-year CR using variables of PE extent, tumor maximum diameter, ETR, age, and KPS using ROC curves are shown in Supplementary Table 1. Only PE extent and ETR showed fair sensitivity and specificity for predicting the 5-year CR at optimal cutoff values around 3 cm and 0.75, respectively. A univariate analysis of median OS and PFS using different cutoff values of PE extent, ETR, and tumor maximum diameter, as well as other common prognostic factors (Li et al. 2011; Mirimanoff et al. 2006; Chaichana et al. 2013; Raysi Dehcordi et al. 2012), is shown in Supplementary Table 2. In the univariate analyses, patients of small PE extent demonstrated relatively long median OS and PFS compared with those of large PE extent. Likewise, patients with small ETR illustrated longer median OS and PFS than those with large ETR. Therefore, by integrating the findings of the statistical significance in univariate analyses, sensitivity, and specificity, we derived the novel combination of PE extent < 3 cm with ETR < 0.75 for LPE and further analysis. Patient characteristics, imaging findings, and treatment modalities according to LPE (positive or negative) are presented in Table 1. Common prognostic factors of KPS (≤ 70 vs. ≥ 80, age < 50 vs. ≥ 50 years, and tumor maximum diameter (< 5 vs. ≥ 5 cm) were examined based on published data (Li et al. 2011; Mirimanoff et al. 2006; Chaichana et al. 2013; Raysi Dehcordi et al. 2012). The distributions of age, KPS, tumor maximum diameter, extent of tumor resection, and chemotherapy regimens were similar between patients with and without LPE.

**Table 1:** Patient characteristics and treatment modalities stratified by limited peritumoral edema status

Characteristics	Item	Total (N = 45)	LPE + (N = 13)	LPE– (N = 32)	*p* value
		N (%)	N (%)	N (%)	
Sex	Female	21 (46.7)	6 (46.2)	15 (46.9)	1.00
	Male	24 (53.3)	7 (53.8)	17 (53.1)	
Age	20–49	15 (33.3)	5 (38.5)	10 (31.2)	0.73
	50–80	30 (66.7)	8 (61.5)	22 (68.8)	
	Mean	54.9	53.5	55.4	0.55
	SD	13.1	12.7	14.5	
KPS	80–100	30 (66.7)	9 (69.2)	21 (65.6)	1.00
	40–70	15 (33.3)	4 (30.8)	11 (34.4)	
Tumor Dmax (cm)	< 5	22 (49.8)	6 (46.2)	16 (50.0)	1.00
	≥ 5	23 (51.1)	7 (53.8)	16 (50.0)	
	Mean	4.8	4.9	4.8	0.86
	SD	1.5	1.8	1.3	
Gross total resection	Yes	30 (66.7)	10 (76.9)	20 (62.5)	0.49
	No	15 (33.3)	3 (23.1)	12 (37.5)	
Chemotherapy	TMZ	22 (48.9)	6 (46.2)	16 (50.0)	1.00
	Nimustine	23 (51.1)	7 (53.8)	16 (50.0)	

*Dmax* maximum diameter, *KPS* Karnofsky performance status, *LPE* limited peritumoral edema, *N* number, *SD* standard deviation, *TMZ* temozolomide

**Fig. 2:**
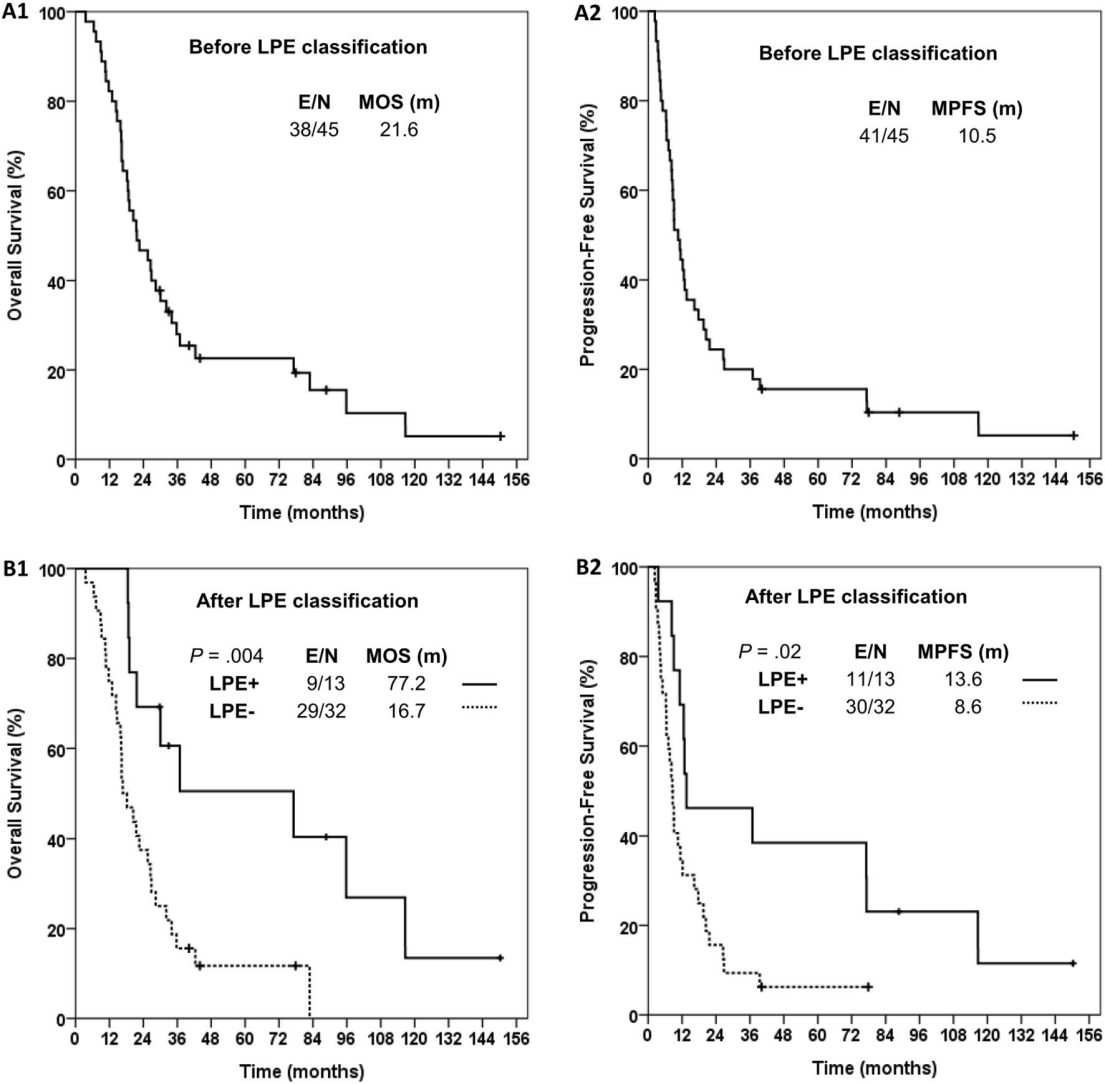
Survival analyses with/without classification of peritumoral edema status. Kaplan–Meier estimates of overall survival and progression-free survival for patients before (A1 and A2) and after (B1 and B2) LPE classification, respectively. *E* event, *LPE* limited peritumoral edema, *MOS* median overall survival, *MPFS* median progression-free survival, *N* number

For all patients (Fig. 2A), the median OS, median PFS, 5-year survival rate, and 5-year CR rate were 21.6 months, 10.5 months, 19.4%, and 13.3%, respectively. After LPE classification, the median OS, median PFS, and 5-year CR rate for cases with LPE vs. without LPE (Fig. 2B) were 77.2 vs. 16.7 (*p* = 0.004), 13.6 vs. 8.6 months (*p* = 0.019), and 38.5% vs. 3.1% (*p* = 0.005), respectively. Results of Cox proportional hazards model analysis for favorable OS and PFS are shown in Table 2. After adjustment for other common prognostic factors, LPE remained as a significant favorable prognostic factor for OS (hazard ratio (HR) 0.29; 95% confidence interval (CI) 0.12–0.69) and PFS (HR 0.42, 95% CI 0.18–0.97).

**Table 2:** Results of Cox proportional hazards model for favorable overall survival and progression-free survival (N = 45)

	OS						PFS					
	Unadjusted			Adjusted^†^			Unadjusted			Adjusted^†^		
Factors	HR	95% CI	*p* value	HR	95% CI	*p* value	HR	95% CI	*p* value	HR	95% CI	*p* value
Age < 50	0.66	0.31–1.41	0.28	0.66	0.28–1.56	0.34	0.74	0.37–1.48	0.4	0.95	0.42–2.17	0.91
KPS ≥ 80	0.99	0.50–1.98	0.98	1.17	0.55–2.50	0.69	0.95	0.49–1.84	0.88	1.02	0.48–2.18	0.96
LPE +	0.30	0.13–0.71	0.006*	0.29	0.12–0.69	0.005*	0.42	0.20–0.88	0.02*	0.42	0.18–0.97	0.04*
Tumor Dmax < 5 cm	1.16	0.63–2.15	0.63	1.55	0.79–3.02	0.2	1.43	0.75–2.71	0.28	1.39	0.74–2.63	0.31
Gross total resection	0.89	0.45–1.78	0.74	1.18	0.55–2.52	0.67	0.81	0.42–1.57	0.53	0.91	0.44–1.87	0.8
Chemotherapy (TMZ)	1.06	0.55–2.07	0.86	0.82	0.41–1.62	0.56	1.36	0.71–2.58	0.36	1.16	0.57–2.33	0.69

*CI* confidence interval, *Dmax* maximum diameter, *HR* hazard ratio, *KPS* Karnofsky performance status, *LPE* limited peritumoral edema, *N* number, *OS* overall survival, *PFS* progression-free survival, *TMZ* temozolomide

*Statistically significant

^†^Adjusted with all variables, including age, KPS, LPE status, tumor maximum diameter, tumor resection extent, and chemotherapy regimen

### Progression patterns after HDPB based on PE status

In progression pattern analysis (Table 3), the rates of 5-year CR, and limited, extended, regional, and distant progressive disease (PD) for patients with LPE vs. without LPE were 38.5% vs. 3.2% (*p* = 0.005), 38.5% vs. 31.3% (*p* = 0.73), 7.7% vs. 40.6% (*p* = 0.04), 7.7% vs. 21.9% (*p* = 0.41), and 0% vs. 34.4% (*p* = 0.02), respectively.

**Table 3:** Progression patterns according to limited peritumoral edema status

PD patterns	Status	Total: 45, N (%)	LPE + : 13, N (%)	LPE–: 32, N (%)	*p* value
5-year CR	Yes	6 (13.3)	5 (38.5)	1 (3.2)	0.005*
	No	39 (86.7)	8 (61.5)	31 (96.8)	
Limited PD	Yes	15 (33.3)	5 (38.5)	10 (31.2)	0.73
	No	30 (66.7)	8 (61.5)	22 (68.8)	
Extended PD	Yes	14 (31.1)	1 (7.7)	13 (40.6)	0.04*
	No	31 (68.9)	12 (92.3)	19 (59.4)	
Regional PD	Yes	8 (17.8)	1 (7.7)	7 (21.9)	0.41
	No	37 (82.2)	12 (92.3)	25 (78.1)	
Distant PD	Yes	11 (24.4)	0 (0)	11 (34.4)	0.02*
	No	34 (75.6)	13 (100)	21 (65.6)	

*CR* complete response, *LPE* limited peritumoral edema, *N* patient number, *PD* progressive disease

*Statistically significant by Fisher’s exact test

**Fig. 3:**
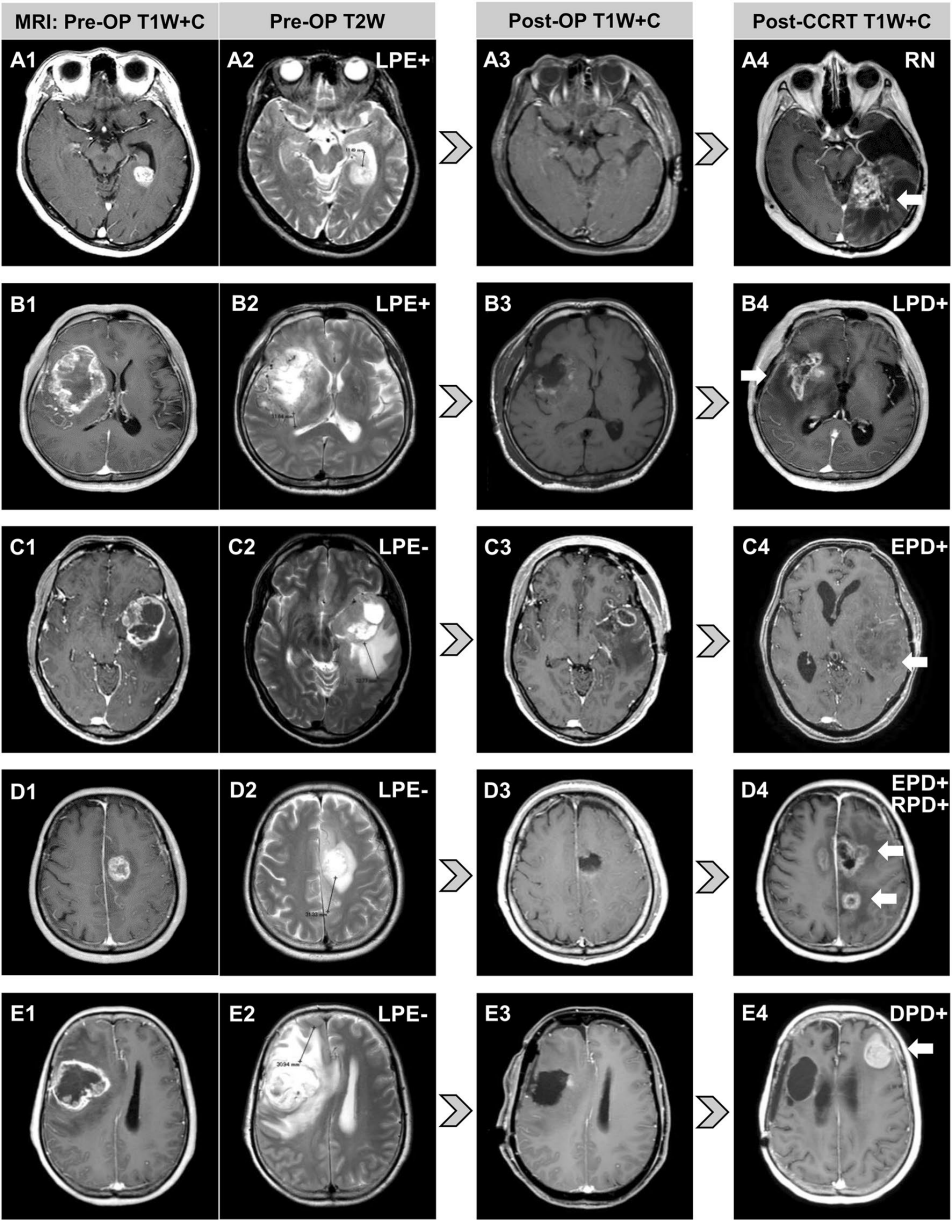
Diverse progression patterns observed after tumor resection and high-dose proton boost for five glioblastoma patients according to their peritumoral edema statuses. The arrows mark tumor progression. **A** LPE + patient with radiation necrosis only. **B** LPE + patient with tumor progression confined to the tumor bed. **C** LPE − patient with EPD. **D** LPE − patient with EPD, extending into the contralateral hemisphere and regional PD. **E** LPE − patient with distant tumor progression at the contralateral frontal lobe. *DPD* distant progressive disease, *EPD* extended progressive disease, *LPE* limited peritumoral edema, *OP* operative, *RN* radiation necrosis, *RPD* regional progressive disease, *T1W* + *C* contrast-enhanced T1-weighted magnetic resonance imaging

Diverse progression patterns evaluated by magnetic resonance imaging (MRI) after tumor resection and HDPB for five cases of glioblastoma with different PE extents are shown in Fig. 3. PE maximum extent and tumor maximum diameter were measured in axial, sagittal or coronal sections. The axial section is shown to facilitate illustration and interpretation. The arrows mark tumor progression. Patient A was LPE + before surgery (Fig. 3A) receiving GTR (Fig. 3A) and had radiation necrosis without tumor progression after HDPB (Fig. 3 A). Patient B was LPE + before surgery (Fig. 3B) receiving GTR (Fig. 3B) and had tumor progression confined to the tumor bed after HDPB (Fig. 3B). Patient C was LPE– before surgery (Fig. 3C) receiving partial resection (Fig. 3C) and had EPD after HDPB (Fig. 3C). Patient D was LPE– before surgery (Fig. 3D) receiving GTR (Fig. 3D) and had EPD to the contralateral hemisphere and regional PD corresponding to the preoperative PE area after HDPB (Fig. 3D). Patient E was LPE– before surgery (Fig. 3E) receiving GTR (Fig. 3E) and had distant tumor progression at the contralateral frontal lobe without local recurrence after HDPB (Fig. 3E). Eleven patients underwent salvage tumor resection due to tumor recurrence. The median survival after salvage surgery (Supplementary Table 3) for patients without and with distant PD was 20.6 and 8.4 months (*p* = 0.005), respectively.

### Toxicities

As our previously published report, for acute toxicities, four patients had headache due to brain edema, which was subsided after corticosteroid administration (Mizumoto et al. 2010). For other acute neurologic toxicities, two patients had seizure of grade 2 (Mizumoto et al. 2016). For late toxicities, six patients developed radiation necrosis without tumor recurrence, which was controlled by necrotomy (n = 5), bevacizumab (n = 1) and both (n = 1), respectively. Among these six patients with radiation necrosis, KPS decreased by 10–30% (Mizumoto et al. 2015).

## Discussion

Several studies have investigated the treatment effects of dose-escalated RT on glioblastoma (Iuchi et al. 2014; Navarria et al. 2017; Shenouda et al. 2017; Tanaka et al. 2005), but there is no clinical index to identify patients with probable long-term disease control after tumor resection followed by high-dose radiation boost. Due to restrictions of RT techniques and clinical trials, the current cohort of 45 patients is a relatively large population to investigate the effects of a radiation dose > 90 Gy for glioblastoma. Patients in this study were treated with a uniform dose 96.6 GyE based on a clinical trial protocol. However, their background features and common prognostic factors had a similar distribution to the general population of patients with glioblastoma (Wegner et al. 2019; Johnson and O’Neill 2012; Wee et al. 2018) (Table 1). Furthermore, the current study was a de novo investigation of glioblastoma patients receiving surgery followed by HDPB, because > 10% of patients were disease-free over 5 years after an exceptionally long period of follow-up.

Using a combination of PE maximum extent with ETR to define LPE, we were able to identify cases of glioblastoma with long-term disease control and those with distinct progression patterns after tumor resection and HDPB, which could have a clinical impact on classifying outcomes after HDPB. To clarify the potential bias in survival analysis and to control for confounding variables, we evaluated the rates of common prognostic factors in LPE + vs. LPE– cases, and found no significant differences (Table 1). Univariate (Supplementary Table 2) and Cox regression (Table 2) survival analyses with the common prognostic factors verified that LPE status was significantly associated with outcome after tumor resection and HDPB. Moreover, we showed that glioblastoma with LPE is significantly associated with a high 5-year CR rate and a low rate of tumor spreading (Table 3), which highlights the potential to personalize future treatment strategies corresponding to LPE status.

### LPE status and survival after HDPB

LPE status provides a clinical index of long-term survival and disease control after glioblastoma resection followed by HDPB. Without biomarker classification, survival in the current study is comparable with that in previous studies using dose-escalated radiotherapy with HDPB (Navarria et al. 2017; Tanaka et al. 2005; Shrieve et al. 1999) (Table 4A). However, the current study has a relatively high 5-year CR rate (13.3% vs. 0–1.6% (Navarria et al. 2017; Tanaka et al. 2005; Shrieve et al. 1999)), indicating that some patients had long-term disease control after tumor resection followed by HDPB. Further analysis revealed that LPE + cases had significantly longer OS and PFS than those without LPE. To prove the reliability of these results, we compared an unadjusted model (single variable) with an adjusted model (six variables) in Cox proportional hazard ratio analysis (Table 2). The HRs and CIs of most variables were similar in the two models, and those for LPE were particularly consistent. These findings suggest that the Cox proportional hazard ratio analysis in the study is reliable.

**Table 4:** Survival and progression patterns using different radiotherapy regimens with and without biomarker classification in the current and published studies

Published study	RT type	RT fractionation, CTV_high_ dose(Gy)/fraction	Combined drugs	Favorable biomarker	Status	Patient No	Survival analysis			PD pattern analysis		
							OS (M)	PFS (M)	5Y-CR rate (%)	LPD rate (%)	EPD rate (%)	DPD rate (%)
A. Dose-escalated RT without biomarker classification												
Tanaka et al (2005)	Photon	HD, 80/40 or 90/45	nimustine + vincristine	–	–	61	19.6	11.1	1.6	–	–	–
Navarria et al (2017)	Photon	HypoF, 60/15	TMZ	–	–	98	16.7	10	{0}	–	–	–
Shrieve et al (1999)	Photon	RT + SRS (6–24 Gy)	–	–	–	78	19.9	–	{10}	–	–	10
Current study	Proton + Photon	HD, 96.6/56, BID	TMZ or nimustine	Before LPE classification	–	45	21.6	10.5	13.3 {19.4}	33.3	31.1	24.4
B. Conventional dose or dose-escalated RT with imaging or molecular biomarker classification												
Liang et al (2017)	Photon	CF, 60/30	TMZ	PE extent < 2 cm	Yes	50	28.6*	17.4^†^	–	–	–	18
					No	86	19.7*	11.0^†^	–	–	–	26
Molenaar et al (2014)	Photon	CF, ~ 78 Gy	Various	*IDH1* mutation	Yes	18	22.0*	6.8^†^	–	–	–	–
					No	80	7.3*	3.8^†^	–	–	–	–
Stupp et al (2009)	Photon	CF, 60/30	TMZ	*MGMT* methylated	Yes	46	23.4*	–	{13.8}	–	–	–
					No	60	12.6*	–	{8.3}	–	–	–
Iuchi et al (2014)	Photon	HypoF, 68/8	TMZ	*MGMT* methylated	Yes	11	36.2*	–	{17.3}	–	–	–
					No	34	14.8*	–	{3.7}	–	–	–
Shenouda et al (2017)	Photon	HypoF, 60/20	TMZ	*MGMT* methylated	Yes	21	53.8*	19.6^†^	0	–	–	–
					No	27	16.2*	8.5^†^	0	–	–	–
Current study	Proton + photon	HD, 96.6/56, BID	TMZ or nimustine	After LPE classification	Yes	13	77.2*	13.6^†^	38.5^§^	38.5	7.7^‖^	0^¶^
					No	32	16.7*	8.6^†^	3.1^§^	31.3	40.6‖	34.4^¶^

{} 5-year survival rate

*5-Y CR* 5-year complete response, *BID* twice a day, *CF* conventional fractionation, *CTV* clinical target volume, *DPD* distant progressive disease, *EPD* extended progressive disease, *ETR* edema to tumor ratio, *HD* high-dose, *HypoF* hypofractionation, *IDH1* isocitrate dehydrogenase 1, *LPD* limited progressive disease, *LPE* limited peritumoral edema, *max* maximum, *MGMT* O^6^-methylguanin–DNA methyltransferase, *OS* overall survival, *PD* progression disease, *PE* peritumoral edema, *PFS* progression-free survival, *RT* radiotherapy, *SRS* stereotactic surgery, *–* data not available

*, †, §, ‖, ¶ Statistically significant

Table 4B lists the comparison of the survival differences after RT of various fractionations and with imaging or molecular biomarker classification, including PE extent, isocitrate dehydrogenase 1 (*IDH1*) mutation, and O^6^-methylguanin-DNA methyltransferase (*MGMT*) promotor methylation, in various studies (Stupp et al. 2009; Liang et al. 2017; Iuchi et al. 2014; Molenaar et al. 2014; Shenouda et al. 2017). The current study shows that LPE status is an effective imaging biomarker for identifying glioblastoma cases that have potential for long-term disease control after tumor resection followed by HDPB. For glioblastomas, large PE extent or PE volume has been associated with a poor prognosis (Liang et al. 2017; Wangaryattawanich et al. 2015).

### LPE status and progression pattern after HDPB

Glioblastoma LPE status provides a clinical index to categorize distinct progression patterns after tumor resection followed by HDPB, which was not reported using molecular biomarker classification (Stupp et al. 2009; Iuchi et al. 2014; Shenouda et al. 2017) (Table 4B). LPE + cases tended to progress confined to the tumor bed (low EPD rate, 7.7%) without distant spread, while LPE– cases tended to progress beyond the tumor bed (EPD rate, 40.6%) or with distant spread (30.4%). These findings demonstrate that glioblastoma cases that are LPE + rather than LPE– tend to reach long-term disease control with a low chance of tumor extension or spreading after tumor resection followed by HDPB, thus confirming our hypothesis. The 5-year CR rate for LPE + cases (38.5%) was significantly higher than that for LPE– cases (3.2%) and is high compared with glioblastoma cases with favorable biomarkers (Stupp et al. 2009; Iuchi et al. 2014; Shenouda et al. 2017) (Table 4B). Moreover, the limited PD rate of LPE + cases (33.3%) was lower than that for published data (72–96.8%) (Brandes et al. 2009; Gebhardt et al. 2014; McDonald et al. 2011), which suggests that LPE + glioblastomas achieved relatively good local control after tumor resection and HDPB.

The extent of tumor resection is associated with prognosis for glioblastomas after conventional dose RT (Li et al. 2011; Mirimanoff et al. 2006). In contrast, patients with GTR vs. non-GTR followed by HDPB had no significant survival difference in the current study, which implies that HDPB can enhance disease control for cases in which GTR is not feasible. For patients with local PD without distant spread, the median survival time after salvage surgery was 20.6 months. Therefore, the long median OS of LPE + cases resulted mainly from long-term CR and partially from salvage surgery. This suggests that reoperation remains as a feasible salvage option after HDPB for cases with local PD only.

### Literature hypotheses for the observations in the study

In the current study, we found that LPE + cases in this study had longer disease control times and less tumor spreading after postoperative HDPB, compared with LPE cases. Herein, we developed hypotheses for our observations based on published literature.

First, long-term disease control without distant spread of LPE + glioblastoma cases after HDPB may be attributable to the treatment effect of HDPB and LPE status. Radiologically, glioblastoma of extensive PE were associated with higher tumor extension and spreading compared with those of limited PE (Liang et al. 2017). Correspondingly, microscopic findings of autopsy brains in patients with glioblastoma demonstrated tumor cells infiltration in the peritumoral edematous area (Yamahara et al. 2010). These findings indicate that PE extent of glioblastoma is correlated with tumor migration or spreading ability, which probably explains why LPE + glioblastoma reached long-term CR without distant spread after tumor resection and HDPB.

Second, glioblastoma patients with *IDH1* mutation and *MGMT* promotor methylation are significantly associated with long-term survival (Stupp et al. 2009; Iuchi et al. 2014; Molenaar et al. 2014; Shenouda et al. 2017). The current study included cases starting from 2001, while *MGMT* promotor methylation and *IDH1* mutation statuses were first introduced to classify glioblastoma prognosis in 2005 (Hegi et al. 2005) and 2009 (Nobusawa et al. 2009), respectively. Therefore, the study protocol did not include collection of data for *IDH1* mutation and *MGMT* promotor methylation statuses. The long-term survival of LPE + glioblastoma cases suggests a potential correlation between LPE + status and *IDH1* mutation or *MGMT* promotor methylation status. Some published studies demonstrated the correlations between glioblastoma imaging and molecular biomarkers (Bangalore Yogananda et al. 2020; Chang et al. 2018a), which supports our hypothesis. A recent study used an MRI-based deep-learning method to classify glioma *IDH* mutation status using preoperative T2-weighted images and obtained a best mean cross-validation accuracy of 97.14% ± 0.04 in predicting *IDH* mutation (Bangalore Yogananda et al. 2020). Another study using preoperative MRI, including T2, FLAIR, and T1-weighted pre- and post-contrast sequences, achieved classification with high accuracy: *IDH1* mutation, 94%, and *MGMT* promotor methylation, 83% (Chang et al. 2018b). These findings illustrate the strong correlation between glioblastoma PE status and *IDH* mutation/*MGMT* promotor methylation.

### Prospective clinical trial design for developing a treatment strategy

**Fig. 4:**
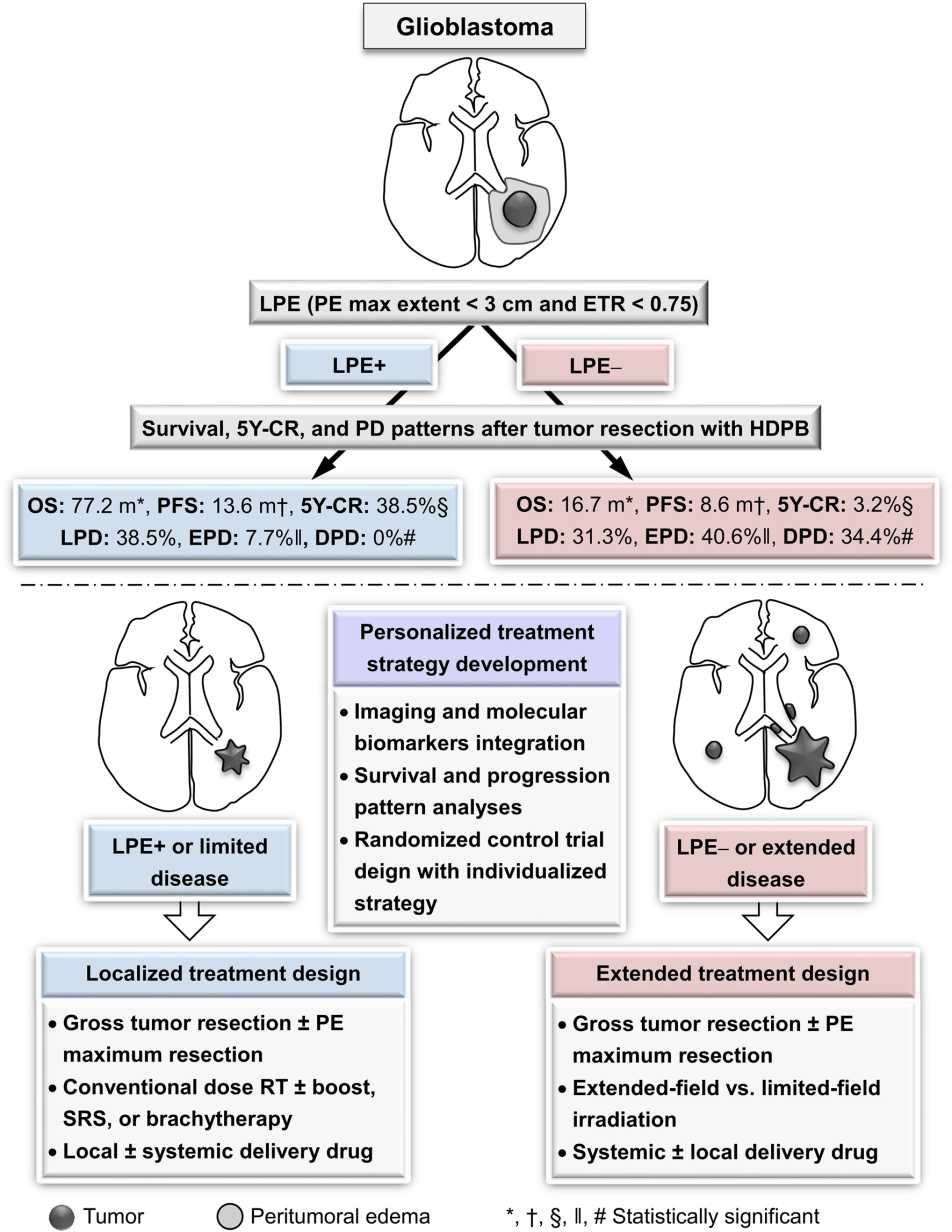
Utilizing peritumoral edema status to facilitate clinical trial design for developing personalized treatment strategies of glioblastoma. *5Y-CR* 5-year complete response, *DPD* distant progressive disease, *EPD* extended progressive disease, *ETR* edema-to-tumor ratio, *HDPB* high-dose proton boost, *LPE* limited peritumoral edema, *OS* overall survival, *PE* peritumoral edema, *PFS* progression-free survival, *SRS* stereotactic radiosurgery

With development of PBT, an index for outcomes following HDPB after tumor resection is needed to develop a corresponding personalized treatment strategy. The major limitation of the current study is the lack of molecular biomarker data. Nonetheless, integration of glioblastoma LPE status and molecular biomarkers to establish a comprehensive index for prognosis grouping can facilitate clinical trial design for investigating individualized treatment strategy (Fig. 4). Since LPE + glioblastomas are significantly associated with a limited progression pattern without distant spread after HDPB, further randomized control trials in LPE + cases are needed to compare the treatment response to conventional dose RT vs. HDPB to verify how HDPB affects the outcome in these cases. Accordingly, development of localized intensive treatment modalities to prevent local recurrence, including stereotactic radiosurgery, intraoperative brachytherapy or local delivery of anticancer agents (Iuchi et al. 2014; Mizumoto et al. 2010; Shenouda et al. 2017; Ashby et al. 2016; Liang et al. 2018; Barbarite et al. 2017), should be mainly investigated for LPE + cases. In contrast, LPE– cases need clinical trials to investigate treatment modalities to reduce tumor spreading, including maximum safe resection of a T1 contrast-enhancing tumor with or without surrounding abnormality in FLAIR images (Li et al. 2016), extended-field irradiation, and use of systemic anticancer agents. The correlation between LPE status and molecular biomarkers requires prospective studies to integrate imaging and molecular biomarker data for optimization of the treatment strategy.

## Conclusions

Based on a comprehensive analysis, we developed an imaging index to classify outcomes of glioblastoma cases after tumor resection followed by HDPB. We also verified our original hypothesis that LPE status can predict long-term disease control and distinct progression patterns of glioblastoma after HDPB, which will help with development of personalized treatment strategies.

## Supplementary Information

Below is the link to the electronic supplementary material.

Supplementary file1 (DOC 65 KB)

## Data Availability

The data sets during and/or analyzed during the current study available from the corresponding author on request.

## Abbreviations

CI: Confidence interval
CR: Complete response
CTV: Clinical target volume
EPD: Extended progressive disease
ETR: Edema-to-tumor ratio
FLAIR: Fluid-attenuated inversion recover
GTV: Gross total resection
HDPB: High-dose proton boost
HR: Hazard ratio
*IDH1*: Isocitrate dehydrogenase 1
KPS: Karnofsky performance status
LPE: Limited peritumoral edema
*MGMT*: O^6^-methylguanin-DNA methyltransferase
MRI: Magnetic resonance imaging
OS: Overall survival
PBT: Proton beam therapy
PD: Progressive disease
PE: Peritumoral edema
PFS: Progression-free survival
RANO: Response Assessment in Neuro-Oncology
RT: Radiotherapy
