# Supplementary material for: Peritumoral edema status of glioblastoma identifies patients reaching long-term disease control with specific progression patterns after tumor resection and high-dose proton boost
Source: J Cancer Res Clin Oncol. 2021 Aug 30;147(12):3503–16. doi: 10.1007/s00432-021-03765-6 (PMC8557163; doi:10.1007/s00432-021-03765-6)
Supplement: Supplementary file 1 — Supplementary file1 (DOC 65 KB) [file 432_2021_3765_MOESM1_ESM.doc]

| **Supplementary Table 1. Sensitivity analysis using receiver operating characteristic**  **curve to discriminate between patients with and without five-year complete response (N = 45)** | | | | | | |
| --- | --- | --- | --- | --- | --- | --- |
| **Factors** | **Area** | **95% CI** | ***p*-Value** | **Optimal cutoff value** | **Sensitivity** | **Specificity** |
| PE extent (cm) | 0.75 | 0.48–1.00 | 0.06 | 2.95 | 0.83 | 0.77 |
| ETR | 0.70 | 0.49–0.91 | 0.13 | 0.74 | 0.83 | 0.32 |
| Tumor Dmax (cm) | 0.55 | 0.48–1.00 | 0.73 | 5 | 0.30 | 0.21 |
| Age | 0.64 | 0.41–0.88 | 0.27 | 50.5 | 0.67 | 0.72 |
| KPS | 0.59 | 0.37–0.80 | 0.50 | 80 | 0.83 | 0.36 |
| **Abbreviations**: CI, confidence interval; Dmax, maximum diameter; ETR, edema-to-tumor ratio; KPS, Karnofsky performance status; PE, peritumoral edema. | | | | | | |

| **Supplementary Table 2. Univariate analysis results for overall survival and progression-free survival**  **using different factors (N = 45)** | | | | | | | |
| --- | --- | --- | --- | --- | --- | --- | --- |
| **Factors** | **Status** | **E/N** | **OS (m)** | ***p*-value** | **E/N** | **PFS (m)** | ***p*-value** |
| PE extent < 3 cm | Yes | 10/14 | 36.9 | 0.009* | 12/14 | 12.8 | 0.04* |
|  | No | 28/31 | 18.2 |  | 29/31 | 8.7 |  |
| ETR < 0.75 | Yes | 23/29 | 28.4 | 0.01* | 26/29 | 11.4 | 0.36 |
|  | No | 15/16 | 16.2 |  | 15/16 | 8.6 |  |
| Tumor Dmax < 5 cm | Yes | 21/22 | 18.5 | 0.28 | 21/22 | 9.1 | 0.63 |
|  | No | 17/23 | 25.6 |  | 20/23 | 11.2 |  |
| LPE (PE < 3 cm and ETR < 0.75) | Yes | 9/13 | 77.2 | 0.004* | 11/13 | 13.6 | 0.02* |
|  | No | 29/32 | 16.7 |  | 30/32 | 8.6 |  |
| **Other common factors** |  |  |  |  |  |  |  |
| Age < 50 | Yes | 13/15 | 19.1 | 0.28 | 13/15 | 10.5 | 0.40 |
|  | No | 25/30 | 21.6 |  | 28/30 | 9.1 |  |
| KPS ≥ 80 | Yes | 25/30 | 18.5 | 0.98 | 27/30 | 9.1 | 0.88 |
|  | No | 13/15 | 22.5 |  | 14/15 | 11.2 |  |
| Gross total resection | Yes | 26/30 | 21.5 | 0.74 | 28/30 | 12.2 | 0.53 |
|  | No | 12/15 | 21.6 |  | 13/15 | 9.1 |  |
| Chemotherapy | TMZ | 18/22 | 22.5 | 0.86 | 21/22 | 10.5 | 0.35 |
|  | Nimustine | 20/23 | 21.5 |  | 20/23 | 9.1 |  |
| **Abbreviations**: Dmax, maximum diameter; E/N, event/number; KPS, Karnofsky performance status; LPE, limited peritumoral edema; OS, overall survival; PE, peritumoral edema; PFS, progression-free survival; TMZ, temozolomide.  *Statistically significant | | | | | | | |

| **Supplementary Table 3. Univariate analysis results for survival after salvage surgery for tumor progression according to distant progressive disease status (N = 11)** | | | |
| --- | --- | --- | --- |
| **Factors** | **E/N** | **OS (m)** | ***p*-value** |
| DPD- | 5/6 | 20.6 | 0.005* |
| DPD+ | 4/5 | 8.4 |  |
| **Abbreviations**: E, event; DPD, distant progressive disease; N, patient number; OS, overall survival.  *Statistically significant | | | |
